# Single centre concept of ‘cold site’ elective surgery during the peak of COVID-19 pandemic : A cohort study

**DOI:** 10.1016/j.amsu.2020.09.047

**Published:** 2020-10-06

**Authors:** Muhammad Rafaih Iqbal, Adeel Abbas Dhahri, Nourelhuda Mohammed Mustafa Darwish, Vardhini Vijay

**Affiliations:** The Princess Alexandra Hospital NHS Trust, Hamstel Road, Harlow, CM20 1QX, UK

**Keywords:** COVID-19, Cold site, Elective surgery

## Abstract

**Objective:**

The COVID-19 pandemic caused a major strain on healthcare systems across the globe. As these systems got overwhelmed with the emergency care of the infected patients, widespread cancellations of elective surgery occurred. Our hospital utilised local private hospital as a dedicated cold site (CS) for urgent elective surgery during the peak of the COVID-19 pandemic. We aim to analyse the outcomes at this dedicated cold site.

**Method:**

A retrospective review of a prospectively maintained database of all the cases operated at the CS during a 2-month period (30 March 2020 to 29 May 2020) was carried out. The primary outcome was 30-day COVID-19 related mortality. The secondary outcomes were 30-day non-COVID-19 related mortality, complications, readmission and development of COVID-19 symptoms.

**Results:**

A total of 153 patients were operated at the CS over the study period with a median age of 57 years (Interquartile range, IQR 47–70). 62% were females and 82% had a Body Mass Index (BMI) less than 30. 73% of the operations were performed for cancer. 59% of the surgeries were graded as intermediate and 26% as major or complex. There was no mortality at 30 days from COVID-19 or non COVID-19 causes. There was only 1 (0.65%) readmission. 7 patients (4.57%) developed complications. 1 (0.65%) patient was diagnosed with COVID-19 in the postoperative period while 3 had COVID-19 symptoms but were tested negative.

**Conclusion:**

Urgent elective surgery is safe and feasible during the COVID-19 pandemic if a dedicated cold site is available.

## Highlights

• This study reports findings of a dedicated cold site elective surgery during the peak of COVID-19 pandemic.
• There was no COVID-19 or non-COVID-19 related 30-day mortality.
• There was only one (0.65%) 30-day readmission and one patient (0.65%) had COVID-19 in the post-operative period.
• Dedicated cold site with multi model approach using careful patient selection and appropriate infection control measures including personal protective equipment is safe and feasible.

## 1 Introduction

The COVID-19 pandemic, since its origin in Wuhan [1], has produced a great strain on the healthcare systems across the globe. There has been a major shift of focus on to emergency patients in order to cope with the surge of acutely ill patients requiring multi-organ support. So as not to overwhelm healthcare systems, all but essential healthcare services were limited and almost all non-emergency surgery came to a halt [2].

It has been estimated that approximately 28 million operations were cancelled or postponed globally during the peak 12 weeks of the pandemic with 2.3 million cancellations per week [3]. This not only has potential disastrous consequences for the individual patient but on the healthcare system as a whole. Patients with cancer are more at risk as a delay in treatment may have a potential impact not only on survival but also on the quality of life [4]. An estimate suggested that globally 2.3 million cancer surgeries have been cancelled or delayed. In the United Kingdom (UK) approximately 36,000 cancer surgeries have been cancelled, clearing this backlog will require a minimum of 11 months with 20% extra activity and would cost approximately £2 billion [3].

There have been some limited reports on increased mortality in patients operated during the COVID-19 pandemic [5]. A mortality of 19% has been reported in a recent study on 278 patients undergoing elective surgery who were diagnosed with COVID-19 peri-operatively [6]. However, a number of multicentre studies are in the process of gathering large volume of data for more conclusive results [7,8].

In order to reduce the impact of this pandemic on urgent elective surgical services at our District General Hospital (DGH), we undertook non-emergency but urgent elective operations at a local private hospital, referred as Cold Site (CS), which was intended to be kept COVID-19 free. We aim to analyse the outcomes of urgent elective surgery at this dedicated CS when the pandemic was at its peak in the community.

## 2 Patients and method

### 2.1 Study design

This was a retrospective review of a prospectively maintained database of consecutive patients undergoing urgent elective surgery at the dedicated CS during the COVID-19 pandemic.

### 2.2 Setting

Our DGH serves a local population of around 500,000 people living in West Essex and East Hertfordshire. During the COVID-19 pandemic, a nearby Private hospital was designated as a dedicated CS after an NHS wide agreement was signed to buy up private capacity during the pandemic. CS has 59 single rooms, 5 operating theatres and the capacity for a 2 bedded Level 2 HDU. A resident medical officer (RMO) is onsite 24/7. Most of the consultants were already working there privately in the pre-covid era, so it seemed feasible and ideal with regards to team work without compromising patient care and safety.

### 2.3 Time period

**Fig. 1:**
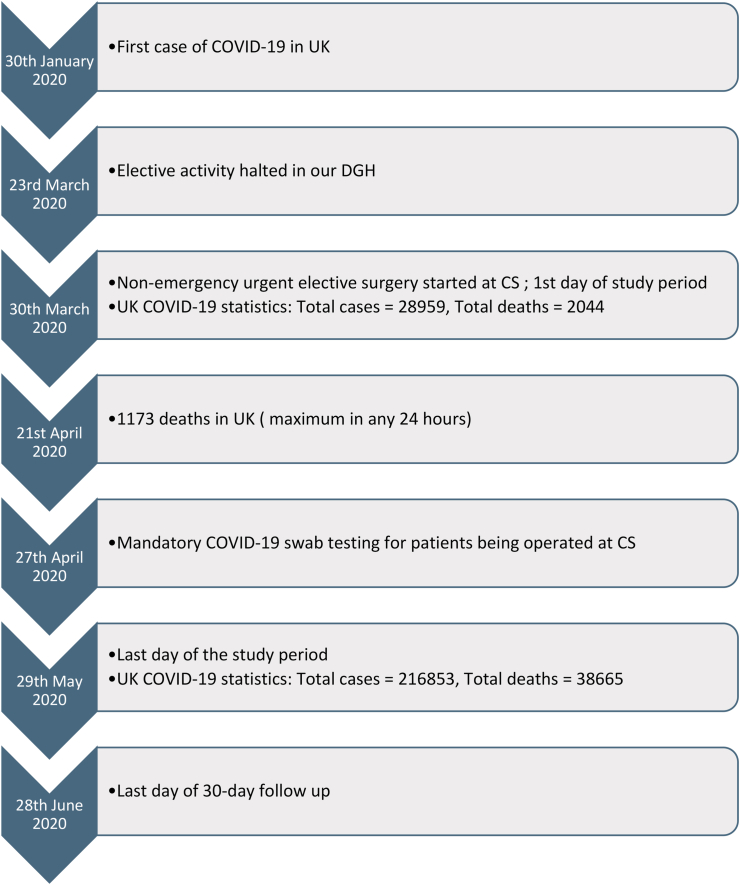
Timeline of events and study period.

2 months (30 March 2020–29 May 2020) (Fig. 1).

### 2.4 Pathway

All patients on the waiting lists of different specialties at the DGH were assessed by individual in-charge consultants and prioritised as per guidance from respective surgical specialty societies [9]. Patients awaiting cancer surgery and those prioritised as requiring urgent non-cancer surgery, then had a notes assessment by a consultant anaesthetist to decide suitability for surgery at the CS. Once they were deemed suitable for operation at the CS, patients were telephoned and offered a date for surgery. Patients willing to attend for surgery were asked to report any COVID-19 related symptoms in the 14 days prior to surgery and to self-isolate for 14 days if feasible. Patients who refused the operation during COVID-19 crisis were excluded from the outcome analysis.

Until 26^th^ April 2020, it was at the discretion of the in-charge consultant to decide if a preoperative chest radiograph (CXR) or Computed Tomography (CT) chest was required before surgery. From 27^th^ April 2020, all patients underwent COVID-19 swab testing 3–5 days before the surgery. Standard personal protective equipment (PPE) were used as per the national recommendations throughout the patient stay at the CS [10,11]. All staff were required to stay away from the hot site (DGH) for 72 h prior to operating at the cold site. Permanent staff at the CS underwent weekly swabbing for COVID-19 and staff moving between the hot and cold sites, underwent swabbing 72 h prior to surgery.

Post-operatively, all patients were reviewed by the operating consultant. Patients were advised to continue to isolate for 2 weeks after surgery. Any patients developing COVID-19 symptoms [12] before discharge were isolated and swab tested for COVID-19. On discharge, patients were given a dedicated contact number to ring if any problems arose. All patients had a telephone consultation at 30 days by a dedicated nurse to check on their progress and if they had any COVID-19 related symptoms [12] (Fig. 1).

### 2.5 Outcome

a. Primary outcome

The primary outcome was 30-day postoperative COVID-19 related mortality.

b. Secondary outcome

The secondary outcomes were 30-day non-COVID-19 related mortality, 30-day postoperative complications and readmission and 30-day development of COVID-19 symptoms.

### 2.6 Data collection

In addition to the prospectively maintained database, hospital electronic records as well as patient notes were reviewed. Data was collected on patient demographics, comorbidities, preoperative COVID-19 status, operative details, length of stay, postoperative COVID-19 symptoms and testing, 30-day readmissions, complications and mortality.

Patient demographics included age and gender. Comorbidities data included hypertension, ischemic heart disease, diabetes mellitus, asthma, chronic obstructive pulmonary disease (COPD), previous history of cancer and current smoking status. Preoperative COVID-19 status data included whether COVID-19 swab was done, swab result, CT scan of the chest, CXR and COVID-19 changes on the CT scan or CXR if present. Operative details included if the surgery was for Cancer Waiting Time (CWT) and operation details. Operation complexity was classified into minor, intermediate, major or complex as per the NICE guidelines [13]. 30-day postoperative data was collected relating to COVID-19 symptoms, COVID-19 swab testing, readmissions, complications and mortality. Complications were classified as per the Clavien-Dindo classification [14]. Mortality data included COVID-19 and non-COVID-19 related mortality.

All the data once collected was independently checked and verified by two authors.

### 2.7 Data analysis

Categorical variables were presented as number and percentage. Continuous variables were presented as median and interquartile range.

### 2.8 Ethical consideration

Hospital NHS research ethics committee exempted this study from ethical approval.

The study has been reported in line with the ‘Strengthening the reporting of cohort studies in surgery’ (STROCSS) criteria [15].

## 3 Results

**Fig. 2:**
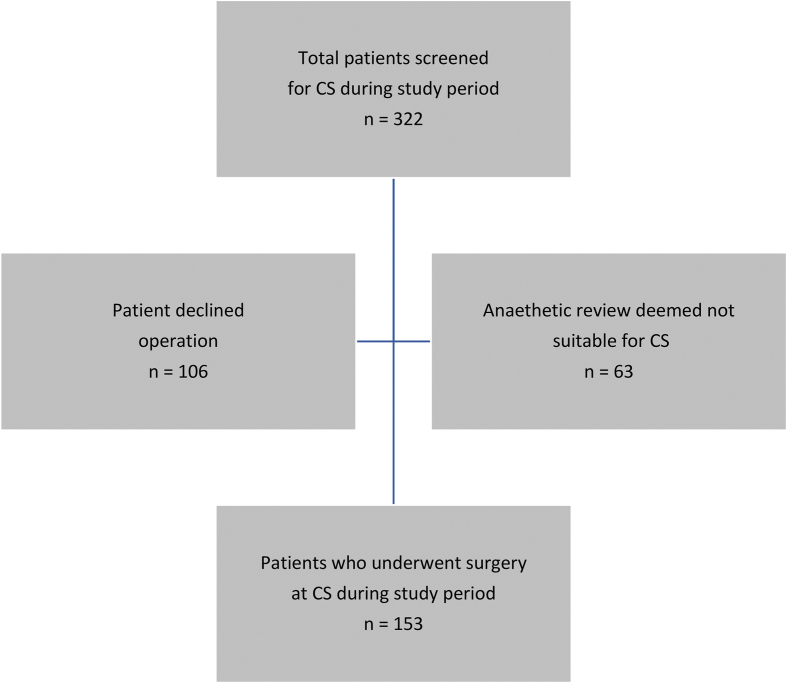
Patient selection.

**Fig. 3:**
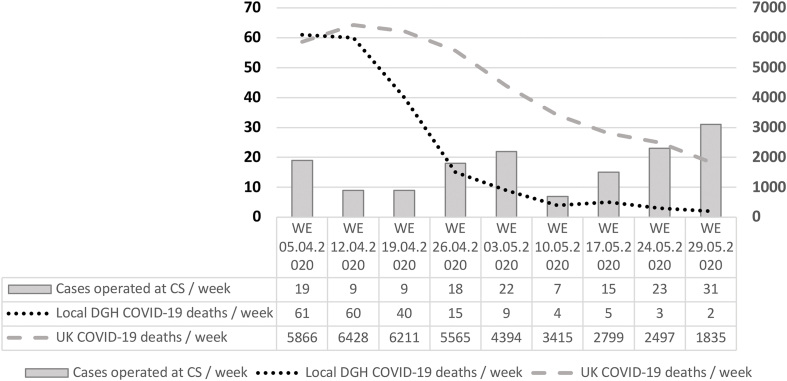
Comparison of cases operated per week at CS with the number of COVID-19 deaths in the local DGH and nationally in UK (WE: week ending).

A total of 322 patients were screened for surgery at the CS. 106 patients (32.91%) declined the operation given the COVID-19 prevalence in the community. 63 patients (19.56%) were deemed not suitable for surgery at CS either due to a BMI >40 or because they required Level 3 care post-operatively. 153 patients (47.51%) underwent surgery at the CS during the study period (Fig. 2). Between 7 and 31 patients were operated weekly at the cold site with more patients refusing to come in for surgery when the pandemic was at its peak locally. This is reflected in low numbers in weeks 2 and 3 (Fig. 3).

Median age of the patients who underwent surgery at CS was 57 years (IQR 47–70). 95 (62.09%) were females. Most of the patients had a BMI <30 (n = 126, 82.35%). 19 patients (12.41%) were active smokers. 13 patients (8.49%) had a previous history of cancer. Most of the patients were American Society of Anaesthesiologists (ASA) grade 1 (n = 72, 47.05%) and grade 2 (n = 78, 50.98%) (Table 1).

**Table 1:** Baseline patient demographics.

Variable	Number n (%)
Median age	57, (IQR 47–70)
Gender	
Male	58 (37.90)
Female	95 (62.09)
BMI	
<30	126 (82.35)
>30	27 (17.64)
Current smoker	19 (12.41)
Co-morbidities	
Diabetes mellitus	17 (11.11)
Ischemic heart disease	8 (5.22)
Hypertension	44 (28.75)
Asthma	13 (8.49)
Chronic obstructive lung disease	5 (3.26)
Previous History of cancer	13 (8.49)
American Society of Anaesthesiologists (ASA) grade	
ASA 1	72 (47.05)
ASA 2	78 (50.98)
ASA 3	3 (1.96)
ASA 4	0 (0)

113 patients (73.85%) were on the Cancer Waiting Time (CWT). Most of the surgeries were classified as Intermediate (n = 91, 59.47%) followed by Major or Complex (n = 40, 26.14%). Median length of stay was 0 days (IQR = 0–1). Most of the cases were day case (n = 100, 65.35%) followed by 1 night stay (n = 44, 28.75%) (Table 2).

**Table 2:** Pre-operative, intra-operative and post-operative data.

	Variable	Number n (%)
Pre-operative	Speciality	
	Breast surgery	36 (23.52)
	General surgery	9 (5.88)
	Urology	48 (31.37)
	ENT	6 (3.92)
	Gynaecology	35 (22.87)
	Maxillo-facial surgery	19 (12.41)
	Cancer Waiting Time (CWT)	
	Yes	113 (73.85)
	No	40 (26.14)
Operative	Anaesthesia	
	General anaesthetic	129 (84.31)
	Local anaesthetic	20 (13.07)
	Spinal anaesthetic	4 (2.61)
	Operation Classification	
	Minor	22 (14.37)
	Intermediate	91 (59.47)
	Major or complex	40 (26.14)
Post-operative	Length of stay (LOS)	
	Day case	100 (65.35)
	1 day	44 (28.75)
	2 days	7 (4.57)
	3 days	2 (1.30)

In the pre-operative period, 83 patients (54.24%) had COVID-19 swab test done and none had a positive result. Chest x-ray was done in 26 (16.99%) and CT-scan of the chest in 1 (0.65%) patients with no positive sign for COVID-19. Post-operatively (within 30 days) 9 patients (5.88%) had COVID-19 swab done with no positive result. Median number of days from surgery to the swab test was 11 days (IQR = 9–16) (Table 3).

**Table 3:** COVID-19 testing.

	Variable	Number n (%)
Pre-operative	COVID-19 swab done	83 (54.24)
	
	COVID-19 positive swab	0 (0)
	Chest X-ray done	26 (16.99)
	
	Positive chest x-ray for COVID-19	0 (0)
	CT-scan Chest done	1 (0.65)
	
	Positive CT-scan chest for COVID-19	0 (0)
Post-operative (within 30-days)	COVID-19 swab done	9 (5.88)
	
	COVID-19 positive swab	0 (0)
	
	Days after surgery (median)	11 (IQR 9–16)

3 patients presented to the Accident and Emergency department at the Hot Site post-operatively. 2 were discharged (one patient with chest pain and the other with urinary retention). One patient was readmitted for an intrabdominal collection which required treatment with intravenous antibiotics. Majority of the complications (n = 6, 3.92%) were Clavien-Dindo grade 2 (Table 4).

**Table 4:** 30-day baseline data.

	Number n (%)
Mortality	
COVID-19 related	0 (0)
Non COVID-19 related	0 (0)
Readmission	1 (0.65)
Complications	7 (4.57)
Clavien-Dindo Complication and Grade	

Grade 1 Urinary retention requiring urinary catheter	1 (0.65)
Grade 2 Wound infection requiring oral antibiotics	3 (1.96)
Intrabdominal collection requiring Intravenous antibiotics	1 (0.65)
Urinary tract infection requiring oral antibiotics	2 (1.30)
Grade 3a	0 (0)
3b	0 (0)
Grade 4a	0 (0)
4b	0 (0)
Grade 5	0 (0)

On the 30-day follow-up call, 15 patients (9.80%) did not respond despite 3 attempts at contact over a week. Of the remaining 138 patients, 4 had symptoms of COVID-19. One of them was picked up on a post-operative staging CT-chest and developed symptoms 1 week after the CT-chest and 3 weeks after the index operation. None of the other three patients tested positive. Median number of days for the onset of symptoms from the surgery was 7 days (IQR = 6.5–9) (Table 5).

**Table 5:** 30-day follow-up COVID-19 symptoms (n=138).

Variable	N (%)
Follow-up COVID-19 symptoms	4 (2.89)

Cough	2 (1.44)
Fever	1 (0.72)
Shortness of breath	1 (0.72)
Loss of smell	1 (0.72)
Loss of taste	1 (0.72)
Body aches	2 (1.44)
Fatigue	2 (1.44)

Days after surgery for onset of symptoms (median)	7 (IQR 6.5–9)

There was no 30-day COVID-19 or non-COVID-19 related mortality.

## 4 Discussion

The results of our study highlight the fact that it was safe and feasible to perform elective surgery at a dedicated CS during the COVID-19 pandemic. This is shown by the fact that there was no COVID-19 or non-COVID-19 related mortality in our study.

Approximately 2.3 million surgeries per week have been cancelled across the globe [3]. This has a potential impact not only on survival especially in cancer patients but also on the quality of life. The first case of COVID-19 in UK was reported on 30^th^ January 2020 [16]. In the UK, the government announced a halt to all non-urgent elective surgeries across the National Health Service (NHS) for 3 months from 15^th^ April 2020 [17]. This was a protective measure as the pandemic was at its peak. Reorganization and leadership were the key. Our DGH stopped all elective activity on 23^rd^ March 2020. All urgent elective operating was then diverted to the CS after careful planning and protocoling to make sure the CS remained COVID-19 free. On 30^th^ March 2020 (1st day of the study period) UK COVID-19 statistics showed a total of 28,959 cases with 2044 deaths while on 29^th^ May 2020 (last day of study period) there were 216,853 with 38,665 deaths [18] suggesting the peak of the pandemic (Figs. 1 and 3).

In the COVID-19 pandemic situation, careful patient selection for surgery is vital. Patients should be fully informed not only about the risks and benefits of surgery but also of the risk of acquiring COVID-19 infection which may lead to mortality. In our study after initial triage, we contacted the eligible patients to see if they were happy to go ahead with their planned operation at a dedicated CS. Risks and benefits were explained. 32.91% (106/322) refused to go ahead with the operation and preferred to wait until the pandemic was over. All patients agreeing for surgery had virtual anaesthetic assessment so that appropriate patients were only selected as per the facilities available. 19.56% (63/322) of the patients were deemed unfit for CS elective surgery (Fig. 2).

Mortality of up to 19% has been reported in patients undergoing elective surgery who were diagnosed with COVID-19 peri-operatively [6]. In our study there was no 30-day mortality from either COVID-19 or Non COVID-19 related causes. Even the 30-day complications and readmission were low (Table 4).

Our follow up was based on the fact that the reported incubation period prior to COVID-19 symptoms has been reported to be 2–14 days with an average of 5.2 days after surgery for the development of 1st symptoms of COVID-19 [5]. An important point to consider is that mandatory COVID-19 testing was only introduced in the 2nd month of the study and despite this there was no mortality and even morbidity was quite low. This further highlights the fact that if a multi model approach of a dedicated cold site, careful patient selection and appropriate infection control measures including personal protective equipment is adopted, then favourable results can still be obtained.

There are limitations to this study including a single centre, retrospective review and a small sample size. During the initial half of the study period, COVID-19 swab testing was limited and thus some asymptomatic infections may have been missed. However, even with these limitations, the results of this study will act a guide when elective surgery returns back to normal.

## 5 Conclusion

This study was carried out during the peak of the COVID-19 pandemic in UK. Results of the study suggest urgent elective surgery is safe and feasible during the COVID-19 pandemic if a dedicated cold site is available. However, overcoming patient stigma about the perceived risks will be important if a second wave was to occur.

## Provenance and peer review

Not commissioned, externally peer reviewed.

## Annals of medicine and surgery

The following information is required for submission. Please note that failure to respond to these questions/statements will mean your submission will be returned. If you have nothing to declare in any of these categories then this should be stated.

## Please state any sources of funding for your research

No funding

## Ethical approval

No ethical approval required.

## Consent

Not applicable.

## Author contribution

Muhammad Rafaih Iqbal: Study conception, Design, Data acquisition, Data analysis and interpretation, Writing – review and editing. Adeel, Abbas Dhahri: Data acquisition, Data analysis. Nourelhuda Mohammed Mustafa Darwish: Data acquisition, Data analysis*.* Vardhini Vijay: Study conception, Design, Data acquisition, Data analysis and interpretation, Writing – review and editing.

## Registration of research studies

1. Name of the registry: Research Registry.

2. Unique Identifying number or registration ID: researchregistry5858.

3. Hyperlink to your specific registration (must be publicly accessible and will be checked): https://www.researchregistry.com/browse-the-registry#home/registrationdetails/5f25fa043e958300185eced6/

## Guarantor

Muhammad Rafaih Iqbal.

## Declaration of competing interest

No conflicts of interest.
